# Lectures on Scarlet Fever

**Published:** 1852-01

**Authors:** 


*Lectures on Scarlet Fever.* By CASPAR MORRIS, M. D., Late Lecturer on Practical Medicine in the Philadelphia Medical Institute, Fellow of the College of Physicians of Philadelphia, &c. &c. Philadelphia : Lindsay & Blakiston, 1851.

These admirable lectures, with which our readers have been favored during the past year, have been separately paged, and neatly bound, and now form a handsome "brochure" upon this deeply interesting subject, which should be in the hands of every practitioner.

